# Stimulation of growth of the human gastric pathogen *Helicobacter pylori *by atmospheric level of oxygen under high carbon dioxide tension

**DOI:** 10.1186/1471-2180-11-96

**Published:** 2011-05-11

**Authors:** Shin Ae Park, Ara Ko, Na Gyong Lee

**Affiliations:** 1 Department of Bioscience and Biotechnology, Sejong University, Seoul 143-747, Republic of Korea

**Keywords:** Helicobacter pylori, growth, atmospheric oxygen level, carbon dioxide

## Abstract

**Background:**

*Helicobacter pylori *(*Hp*), a human pathogen that is associated with gastritis, peptic ulcer, and gastric cancer, has been considered a microaerophile, but there is no general consensus about its specific O_2 _requirements. A clear understanding of *Hp *physiology is needed to elucidate the pathogenic mechanism(s) of *Hp *infection.

**Results:**

We cultured *Hp *under a range of O_2 _levels with or without 10% CO_2 _and evaluated growth profiles, morphology, intracellular pH, and energy metabolism. We found that, in the presence of 10% CO_2_, the normal atmospheric level of O_2 _inhibited *Hp *growth at low density but stimulated growth at a higher density. Field emission scanning electron microscopy and fluorescence microscopy of *Hp *cells cultured under 20% O_2 _tension revealed live spiral-shaped bacteria with outer membrane vesicles on a rugged cell surface, which became smooth during the stationary phase. Fermentation products including acetate, lactate, and succinate were detected in cell culture media grown under microaerobic conditions, but not under the aerobic condition. CO_2 _deprivation for less than 24 h did not markedly change cytoplasmic or periplasmic pH, suggesting that cellular pH homeostasis alone cannot account for the capnophilic nature of *Hp*. Further, CO_2 _deprivation significantly increased intracellular levels of ppGpp and ATP but significantly decreased cellular mRNA levels, suggesting induction of the stringent response.

**Conclusions:**

We conclude, unlike previous reports, that *H. pylori *may be a capnophilic aerobe whose growth is promoted by atmospheric oxygen levels in the presence of 10% CO_2_. Our data also suggest that buffering of intracellular pH alone cannot account for the CO_2 _requirement of *H. pylori *and that CO_2 _deprivation initiates the stringent response in *H. pylori*. Our findings may provide new insight into the physiology of this fastidious human pathogen.

## Background

Oxygen is important for many organisms; because of its high redox potential, it is a common electron acceptor in cellular respiration. However, diverse metabolic reactions generate cell-damaging reactive oxygen species such as superoxide (O_2_^-^) and hydrogen peroxide as byproducts. In response, cells have developed oxidative stress defense systems to protect themselves from oxidative damage. Microorganisms are classified into three large categories--aerobic, anaerobic, and microaerophilic--on the basis of their ability to use oxygen as an electron acceptor during ATP generation. Microaerophiles show optimal growth at 2% to 10% O_2_, but cannot survive under the normal atmospheric level of O_2 _[1].

*Helicobacter pylori *(*Hp*) is a gram-negative human pathogen that resides in the mucus layer of the stomach. It affects more than half of the world's population and is often associated with gastritis, peptic ulcer, and gastric cancer [2,3]. Numerous studies have shown that *Hp *uses both aerobic respiration and fermentation pathways. Complete genome sequencing and studies of *Hp *metabolism and physiology indicate that *Hp *uses glucose as its primary energy and carbon source by the Entner-Doudoroff and pentose phosphate pathways [4-9]. Depending on culture conditions, *Hp *anaerobically produces lactate and acetate from pyruvate or aerobically produces acetate or CO_2 _[4,7,10,11]. *Hp *metabolizes pyruvate by the anaerobic mixed acid fermentation pathway, accumulating alanine, lactate, acetate, formate, and succinate [12]. It also uses the tricarboxylic acid cycle, which appears to be a noncyclic, branched pathway characteristic of anaerobic metabolism that produces succinate in the reductive dicarboxylic acid branch and α-ketoglutarate in the oxidative tricarboxylic acid branch [13]. *Hp *constitutively expresses the aerobic respiratory chain with a *cbb*3-type cytochrome c oxidase as the terminal oxidase [14]. Whole genome analysis of two *Hp *strains revealed the presence of genes encoding components of the membrane-embedded F0 proton channel and the catalytic F1 complex, suggesting that *Hp *produces a significant portion of its ATP by aerobic respiration [9,15]. In addition, *Hp *uses anaerobic respiration utilizing H_2 _as an electron donor [16].

Since its discovery in 1984, *Hp *has been considered a microaerophilic bacterium highly susceptible to environmental O_2 _tension [17]. *Hp *is a spiral-shaped bacillus that, when exposed to a high O_2 _concentration, converts to a full coccoid form that is viable but nonculturable [18,19]. *Hp *is generally cultured under microaerobic conditions using a GasPak or CO_2 _chamber to achieve adequate growth, and its cultivation can be difficult and cumbersome [20]. Therefore, significant efforts have been made to increase the efficiency of *Hp *cultivation [21-23].

There are many hypotheses for the microaerophilic requirements of bacteria: high sensitivity to toxic forms of oxygen present in the culture medium, excessive metabolic generation of toxic forms of oxygen, low respiratory rates, iron deficiency, lack of protective enzymes, unusually oxygen-sensitive cell constituents, and reliance on oxygen-labile substrates (see reference [24] for review). The antioxidant defense system of *Hp *has been studied extensively because of its unique microaerophilic nature and clinical importance. *Hp *has been found to express oxidative stress resistance enzymes including superoxide dismutase (SodB), catalase (KatA), as well as peroxiredoxins, alkyl hydroxide reductases, bacterioferritin co-migratory protein and thiol peroxidase (see reference [25] for review). In addition, *Hp *expresses neutrophil-activating protein (NapA), which protects cells from oxidative stress damage, DNA repair proteins (Nth, MutS, RuvC), an oxidized protein repair system (Msr), and the thioredoxin system (thioredoxin and thioredoxin reductase) [25]. Despite these diverse antioxidant systems, *Hp *remains vulnerable to the toxicity of environmental levels of oxygen. Several lines of evidence have suggested that *Hp *may not be microaerophilic. *Hp *strains exhibit a range of susceptibility to high O_2 _tension, and two strains adapted to aerobic growth have been isolated [26]. In addition, researchers, including our group, routinely culture *Hp *strains in regular incubators supplied with 5% to 10% CO_2 _[27-30]. Bury-Moné et al. recently reported that at a high cell density and in the presence of 5% CO_2_, *Hp *showed similar growth profiles in liquid cultures under microaerobic and aerobic conditions, suggesting that *Hp *may not be microaerophilic [31].

Despite the clinical importance and extensive studies of *Hp*, many basic aspects of its microaerophilicity remain unclear. To extend our knowledge of its pathogenesis in host environments, we must first elucidate its response to O_2 _to characterize its physiology and energy metabolism. In the present study, we assessed the response to O_2 _and demonstrated that *Hp *growth is promoted by atmospheric O_2 _levels when inoculated at high density under 10% CO_2_. Our data also suggest that buffering of intracellular pH alone cannot completely explain the CO_2 _requirement of *Hp*. Our finding that there is no need to control O_2 _tension for *Hp *cultivation at a high cell density may make it substantially easier for researchers to perform experiments with this fastidious pathogen.

## Methods

### *Hp *strains and culture conditions

The *Hp *strain 26695 was purchased from American Type Culture Collection (Manassas, VA, USA) and also provided by Dr. A. van Vliet of Erasmus MC University, The Netherlands. Strain SS1 was provided by Dr. Y. H. Choe of Samsung Medical Center, Seoul, Korea, and strains 1061 and 11638 by Dr. A. van Vliet. *Hp *clinical strains G9 and A16 were isolated from antral biopsy specimens of Korean adolescents with gastritis and iron deficiency anemia, respectively. They were analyzed and published previously [30], and re-analyzed for this study.

After revival from frozen stocks, the bacteria were pre-cultured for 24 to 48 h on Brucella broth (BB; Difco, Sparks, MD, USA) agar plates containing 10% horse serum (Gibco BRL, Life Technologies, Rockville, MD, USA) at 37°C in an incubator under 10% CO_2 _or in a microaerobic jar (CampyGen gas packs, Oxoid, Hampshire, England). For experiments, cultured cells were collected from the agar plates, washed, and resuspended in BB liquid medium, and then inoculated to the desired optical density at 600 nm (OD_600_) into BB liquid medium buffered with 10 mM sodium phosphate (pH 6.3) and supplemented with 10% new born calf serum (NBCS). Then, 20-ml aliquots were distributed into 100-ml flasks, which were filled with gas mixtures containing a range of O_2 _(0%, 5% or 20%) in the absence or presence of 10% CO_2_. The actual O_2 _levels in the culture flasks filled with gas mixtures were 2%, 8%, and 20%, respectively, as determined by Oxygen Indicator XP-3180 (New Cosmos Electric, Osaka, Japan). Bacterial cultures were incubated at 37°C with shaking at 200 rpm.

### Determination of bacterial growth profiles

*Hp *cells collected from agar plates were washed and inoculated into BB-NBCS (OD_600_, 0.1). Then, 20-ml aliquots were inoculated into 100-ml flasks, and cultured under various gas conditions. An aliquot of each culture was taken at 6, 12, 24, 36, 48, and 60 h, and the OD_600 _and pH of the culture media were determined. The flasks were then filled with the appropriate gas mixtures and incubated further. These experiments were repeated without exposure to atmospheric O_2_; 15 flasks were inoculated with *Hp *and cultured under various gas conditions. One flask was taken to measure OD_600 _and media pH at each time point.

To determine effect of different gas conditions on cell viability, each culture was serially diluted 10-fold with BB liquid medium, and 100-μl aliquots were spread on BB agar plates supplemented with 10% horse serum. The plates were incubated at 37°C under 10% CO_2 _atmosphere for 3 to 6 days, and the colonies were counted.

### Field emission-scanning electron microscopy

*Hp *cultured in liquid media were harvested by centrifugation, pre-fixed in 2% paraformaldehyde and 2% glutaraldehyde in a 0.1 M phosphate buffer (pH 7.2) for 2 h at 4°C, and then post-fixed in 1% osmium tetroxide at 4°C for 2 h. The specimens were dehydrated with a series of ethanol solutions (30%-100%) and treated with hexamethyldisilazane twice for 15 min. The specimens were mounted on metal stubs, coated with a thin layer platinum under argon using a sputter-coater (SCD 005; BAL-TEC, Bannockburn, IL, USA), and then visualized by field emission-scanning electron microscopy (FE-SEM) (Supra 55VP; Carl Zeiss, Oberkochen, Germany) at the accelerating voltage of 2 kV at the National Instrumentation Center for Environmental Management (NICEM; Seoul, Korea). Images were captured in TIFF format.

### Confocal microscopy

To determine membrane integrity, bacterial cells were stained with membrane-permeant and -impermeant fluorescent dyes according to the manufacturer's instructions (Live/Dead BacLight Bacterial Viability Kit; Molecular Probes, Eugene, OR, USA) followed by confocal microscopy. *Hp *cells from BB agar plates were inoculated (OD_600_, 0.01 or 0.1) into BB-NBCS media and grown under various gas conditions. Aliquots were taken at 12 or 36 h, stained with SYTO 9 and propidium iodide (PI) for 15 min, and washed twice with phosphate buffered saline (PBS). Cells were then spread on slide glasses, covered with mounting medium and cover slips, and visualized by confocal microscopy (Leica TCS SP5; Leica Microsystems GmbH, Wetzlar, Germany). SYTO 9 is a green fluorescent membrane-permeant dye that labels all bacteria by staining nucleic acid, whereas PI is a red fluorescent membrane-impermeant dye that labels only bacteria with damaged membranes.

### High performance liquid chromatography analysis of organic acid metabolites

The concentrations of fermentation products in the *Hp *culture media were determined by high performance liquid chromatography (HPLC) using the HP1100 system (Hewlett Packard, Palo Alto, CA, USA) at NICEM. *Hp *cells grown on agar plates were collected, washed, and inoculated into 20 ml of fresh media (OD_600_, 0.1). Cells were cultured under various gas conditions for 36 h, and the culture medium was collected and divided into two aliquots (one of which was spiked with 15 mM pyruvate as internal control for quantification), which were processed simultaneously. The culture medium was extracted twice with phenol/chloroform to remove proteins and then passed through a 0.45-μm syringe filter. The samples were injected into an ion exchange column (Aminex HPX-87H, 300 × 7.8 mm; Bio-Rad, Richmond, CA, USA), and eluted at 40°C with 0.01 N H_2_SO_4 _at a flow rate of 0.5 ml/min. Organic acids were analyzed with a refractive index detector HP1100 (Hewlett Packard). Solutions containing glucose and organic acids including acetate, formate, propionate, lactate, pyruvate, succinate, and butyrate were used as standards. Organic acids were identified by their retention times, and the levels of glucose and organic acids in each sample were determined by their peak areas. Results were normalized against the spiked pyruvate, and the amount of secreted organic acid per mg bacterial protein was calculated.

### Fluorimetric analysis of cytoplasmic and periplasmic pH

The cytoplasmic and periplasmic pH of *Hp *cells was determined with fluorescent dyes. Bacterial cells grown on BB agar plates were harvested, washed, and inoculated into 20 ml of fresh BB-NBCS media (OD_600_, 0.05). To measure cytoplasmic pH, the membrane-permeant pH-sensitive fluorescent probe, 2,7-bis-(2-carboxyethyl)-5-carboxyfluorescein acetoxymethyl ester (BCECF-AM; Molecular Probes) was added to the culture media (final concentration, 10 μM). To measure periplasmic pH, we used 2,7-bis-(2-carboxyethyl)-5-carboxyfluorescein (BCECF, Molecular Probes), which penetrates the outer membrane but not the inner membrane. The cells were grown at 37°C with shaking at 200 rpm under aerobic conditions in the presence or absence of CO_2 _(O_2_:CO_2_:N_2 _= 20%:10%:70% or 20%:0%:80%, v/v/v). An aliquot of each culture was taken at 0.5, 3, 6, 12, 24, 36, and 60 h, and the cells were analyzed with a FACSCalibur flow cytometer (Becton Dickinson, San Jose, CA, USA). Acquisition and analysis of samples was performed with CELLQuest Pro software (Becton Dickinson).

### Luciferase assay of intracellular ATP

*Hp *grown in BB-NBCS liquid media were harvested at mid-log phase, washed, and inoculated into 20 ml of fresh media (OD_600_, 0.3). Rifampicin was added to the culture medium at the final concentration of 300 μg/ml. The flasks were then filled with various gas mixtures and incubated at 37°C for 0.5 or 2 h. Cells were then harvested and washed with 0.1 M Tris⋅Cl buffer (pH 7.75) containing 2 mM EDTA. The cell pellets were resuspended and lysed by sonication on ice with an ultrasonic processor (VC505; Sonics and Materials, Newton, CT, USA). Lysates were centrifuged at 13,600 × *g *at 4°C for 3 min. For the luciferase assay, 250 μl of the *Hp *lysate (supernatant fraction) was mixed with 25 μl firefly lantern extract (Sigma, St. Louis, MO, USA), and luminescence was determined with the Infinite M200 Microplate Luminescence Reader (TECAN, Männedorf, Switzerland). The ATP content of the bacterial lysate was determined with an ATP standard curve and converted into nanomoles of ATP per mg bacterial protein.

### HPLC determination of intracellular nucleotides

Intracellular nucleotide, purine, and pyrimidine levels were determined by HPLC using the method described by Huang et al. with slight modifications [32]. *Hp *grown in BB-NBCS liquid media was harvested at mid-log phase, washed, and inoculated into 20 ml of fresh medium (OD_600_, 0.3). The cells were cultured for 1 h under 20% O_2 _tension in the absence or presence of CO_2_. Bacterial cells were then harvested, washed with ice-cold PBS, and divided into two aliquots (one of which was spiked with 50 μM dTTP as an internal control for quantification), which were processed simultaneously. The cell pellets were resuspended in 50 μl of 6% trichloroacetic acid, vortexed for 20 seconds, and kept on ice for 10 min. These cell extracts were then centrifuged at 13,600 × *g *at 4°C for 10 min. The supernatants were mixed with 150 μl of 1 M Tris⋅Cl (pH 7.5) and maintained -70°C. HPLC analysis was performed with the HP1100 system (Hewlett Packard) at the Seoul Center of the Korea Basic Science Institute (Seoul, Korea). Samples (70 μl) were injected into the Vydac column (4.6 × 250 mm; Agilent, Santa Clara, CA, USA) and eluted at room temperature at a flow rate of 1 ml/min. The mobile phase consisted of a gradient of buffer A [0.1 M KH_2_PO_4_, 5 mM tetrabutylammonium hydrogen sulfate, 2.5% (v/v) acetonitrile, pH 6.0] and buffer B [0.1 M KH_2_PO_4_, 5 mM tetrabutylammonium hydrogen sulfate, 25% (v/v) acetonitril, pH5.5]. Nucleotides and bases were detected with a UV detector and identified by retention time relative to the standards. The levels of nucleotides and bases in each sample were determined by comparison with a standard curve. The following were used as standards for analysis: adenine, guanine, cytosine, thymine, uracil, ATP, GTP, CTP, UTP, UMP (Sigma), dATP, dGTP, dCTP, dTTP (Takara Korea, Seoul, Korea), ppGpp, and pppGpp (Santa Cruz Biotechnology, Santa Cruz, CA, USA). Results were normalized using the levels of the spiked dTTP, and the nanomoles of intracellular nucleotides and bases per mg bacterial protein was calculated.

### Statistical analysis

Groups were compared by Student's *t*-test. *P *values less than 0.05 were considered significant. Results are expressed as mean ± standard deviation (SD).

## Results

### Atmospheric level of O_2 _induces *Hp *growth under high CO_2 _tension

To evaluate the effects of O_2 _on *Hp *growth, we grew *Hp *strain 26695 in liquid medium under various gas conditions and determined growth profiles by measuring OD_600_. Our preliminary studies showed that the culture medium pH rapidly rose as cell density increased, subsequently inhibiting growth as described previously [33]. However, the culture medium pH was lower in cultures exposed to 10% CO_2 _than in the absence of CO_2_. To eliminate the effect of pH on *Hp *growth, we buffered the BB-NBCS medium for all experiments in the present study with sodium phosphate to pH 6.3, which is the *pK*_*a *_value for the bicarbonate and carbonic acid reaction. Starting cultures used for experiments were prepared in a same way throughout the study as described in Materials and Methods. We observed that more than 99% of cells in the starting cultures were membrane-intact after Live/Dead membrane permeability staining and that more than 80 percent of the cells were viable.

**Figure 1:**
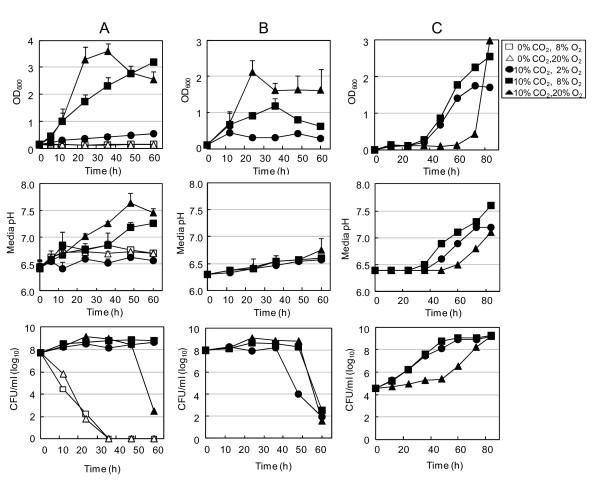
**Atmospheric level of O_2 _stimulates *Hp *growth in the presence of CO_2_**. *Hp *26695 cells collected from agar plates were inoculated into BB-NBCS at 5 × 10^7 ^CFU/ml (A and B) or 3 × 10^4 ^CFU/ml (C) and cultured under 2%, 8%, or 20% O_2 _tension in the absence or presence of 10% CO_2_. An aliquot of each culture was taken at the indicated time points to determine absorbance at 600 nm, culture media pH, and viable cell counts. For data shown in A and C, each flask was refilled with the appropriate gas mixture and incubated for measurements at later time points. For data shown in B, 15 flasks were inoculated with the preculture, filled with mixed gas, and incubated. One flask was used at each time point for measurements; flasks were used only once to prevent exposure of cultures to atmospheric oxygen. Absorbance at 600 nm and media pH data shown in A and C are expressed as mean ± SD of triplicate cultures and are representative of ten and three experiments, respectively. Data shown in B are mean ± SD of four independent experiments. Colony counting data are representative of four independent experiments with similar results.

In contrast to previous reports, we observed that *Hp *grew faster and to a higher density under 20% O_2 _tension than under 8% O_2 _tension in the presence of 10% CO_2 _(Figure 1A). Under 20% O_2_, growth peaked at 36 h and then declined. Very little growth was detected in the culture grown under 2% O_2 _tension, and no growth was detected in the absence of CO_2 _regardless of O_2 _concentration. The culture medium pH increased in parallel with bacterial growth, indicating ammonia production by growing bacteria (Figure 1A). Viable cell count analysis also revealed that the number of cells in aerobic cultures was 3-4 times higher than that in microaerobic cultures at 24 h, but rapidly decreased after 48 h. In contrast, a rapid drop in viable cell count was observed in cultures grown without CO_2_, and no viable cells were detected at 36 h. In this first experiment, we took measurements from aliquots obtained from the culture flasks at each time point; the flasks were then refilled with the appropriate gas mixtures and incubated further for subsequent analysis. As a result, cultures grown under 2% or 8% O_2 _tension were exposed to atmospheric oxygen during sampling, which may have affected results.

To verify our results, we inoculated 15 flasks with a preculture, filled with the appropriate gas mixtures, and incubated. At each time point, we measured the bacterial growth and culture medium pH of one flask of each gas condition. Flasks were sampled only once to prevent exposure of cultures to atmospheric O_2_. The growth profiles were similar to those presented in Figure 1A, but absorbance values were generally lower and culture medium pH increased only modestly (Figure 1B). However, without periodic exposure to atmospheric O_2_, *Hp *growth was much lower under 8% O_2 _tension. These results confirmed that 20% O_2 _does not kill *Hp *but increases growth compared with 2% or 8% O_2_.

Bury-Moné et al. reported that *Hp *lost its microaerophilic properties, demonstrating similar growth profiles under 5% and 21% O_2 _tension when inoculated at a high cell density but not at low density [31]. In the present study, we inoculated cells to an OD_600 _of 0.1, corresponding to 5 × 10^7 ^CFU/ml. We also inoculated BB-NBCS with a preculture containing 5 × 10^6 ^CFU/ml and cultured under 2%, 8%, or 20% O_2 _tension in the presence of 10% CO_2_, and obtained similar results (data not shown). At 12 h, the bacterial concentration was slightly lower under 20% O_2 _tension than under 8% O_2_; this was observed at 6 h in the cultures inoculated at higher cell density. We further reduced the inoculum to 3 × 10^4 ^CFU/ml, which resulted in prolonged lag periods in all three cultures. In particular, cultures grown under 20% O_2 _showed barely detectable growth until 48 h, but subsequently grew exponentially (Figure 1C). In this experiment, we replenished flasks with the appropriate gas mixtures every 12 h; thus, decreased O_2 _levels may not be the reason for rapid growth at high density. Gram-stain analysis and viable cell counts showed that this apparent lack of growth was not due to coccoid formation or cell death. Increases in medium pH were consistent with the growth profiles of the cultures. Taken together, these results suggest that high O_2 _tension inhibits growth of cultures inoculated at low density but increases growth of cultures inoculated at higher density.

To confirm these results, we compared the growth profiles of other *Hp *strains incubated under 8% and 20% O_2 _tension. *Hp *strains SS1 and 1061 also grew more quickly under 20% O_2 _tension (data not shown). Because these laboratory strains may have adapted to high O_2 _tension after many in vitro passages, we also tested the clinical strains G9 and A16 and obtained similar results (data not shown). Growth of all *Hp *strains tested, other than strain 1061, rapidly declined when the medium pH reached approximately 7.3, demonstrating the high sensitivity of *Hp *to alkaline pH. To verify that the ability of *Hp *cells to grow under 20% O_2 _tension is not due to adaptation to atmospheric O_2 _tension, we also determined the growth (both low-density and high-density) of strains 26695 and 11638, which had been maintained under only microaerobic conditions, and obtained similar results (data not shown). On the basis of these results, we concluded that atmospheric levels of O_2 _do not kill *Hp *but rather promote growth at high cell densities.

**Figure 2:**
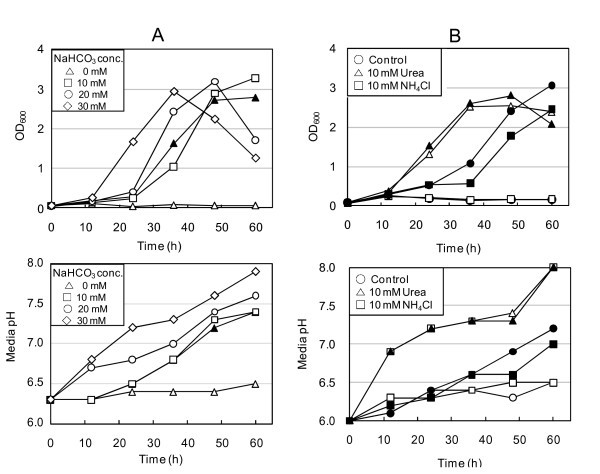
**Bicarbonate and urea support *Hp *growth in place of CO_2_**. *Hp *26695 cells harvested from agar plates were inoculated into BB-NBCS supplemented with NaHCO_3 _(A), urea or NH_4_Cl (B) and cultured under 20% O_2 _tension in the absence (open symbol) or presence (closed symbol) of 10% CO_2_. At each time point, an aliquot of each culture was taken to determine growth and culture medium pH. Data shown in A and B are representative of five and two independent experiments, respectively.

Because CO_2 _is essential for *Hp *growth, we assessed the ability of bicarbonate to substitute for CO_2 _in supporting *Hp *growth. *Hp *cells were cultured in BB-NBCS supplemented without or with sodium bicarbonate (10, 20, or 30 mM) under 20% O_2 _in the absence of CO_2_. Growth was proportional to bicarbonate concentration, indicating that *Hp *can utilize bicarbonate in place of CO_2 _(Figure 2A). Cultures grown with higher bicarbonate levels reached a growth peak at the time point at which medium pH was approximately 7.3. Thus, the early entry of these cultures into the stationary phase appeared to be due to high culture medium pH.

To survive in the highly acidic host environment, *Hp *contains the enzyme urease, which converts urea to ammonia and CO_2 _[34-38]. Urea supports *Hp *growth in the absence of CO_2 _only at acidic pH levels; the CO_2 _generated from urea plays a role in periplasmic and cytoplasmic buffering [39,40]. We tested the possibility that CO_2 _generated from urea was sufficient to support the growth of *Hp*. We buffered culture medium (pH 6.3) to prevent high pH from inhibiting *Hp *growth. In the absence of CO_2_, urea markedly shortened the lag phase of growth, but combining urea with CO_2 _did not yield additive effects on growth (Figure 2B). We also cultured *Hp *in the medium supplemented with NH_4_Cl in the absence or presence of CO_2_. NH_4_Cl supply did not support *Hp *growth in the absence of CO_2 _nor shortened the lag period in the presence of CO_2_, excluding the possibility that ammonium produced from urea supports *Hp *growth.

Supplementation of the culture medium with oxaloacetate, which is rapidly converted into pyruvate and CO_2_, also supported *Hp *growth in the absence of CO_2_, but addition of oxaloacetate to cultures incubated under 10% CO_2 _did not increase *Hp *growth (data not shown). In contrast, pyruvate supplementation could not substitute for CO_2 _(data not shown). Taken together, these data demonstrate the CO_2 _requirement of *Hp *for optimal growth and its ability to utilize bicarbonate in place of CO_2_.

### Lack of CO_2 _but not high O_2 _tension transforms *Hp *into the coccoid form

**Figure 3:**
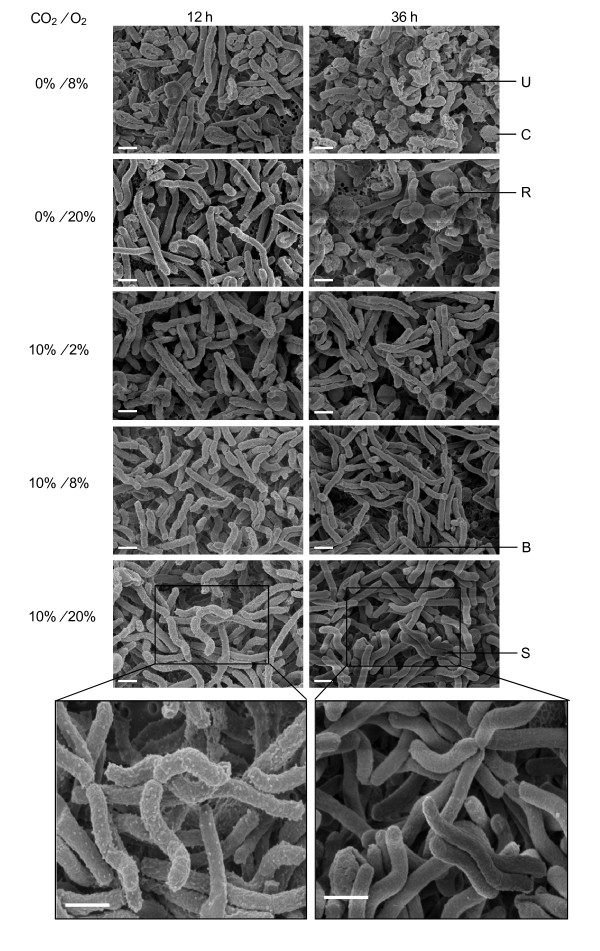
**FE-SEM images reveal healthy spiral morphology of *Hp *cells cultured under aerobic condition**. *Hp *26695 was cultured in liquid medium with shaking under 2%, 8%, or 20% O_2 _tension in the absence or presence of 10% CO_2_. Cells harvested at 12 or 36 h were visualized by FE-SEM. Examples of spiral (S), bacillary (B), U-shaped (U), rounded (R), and coccoid (C) forms are indicated. In enlarged pictures, outer membrane vesicles can be seen on cells cultured under 20% O_2 _tension for 12 h, but not cells cultured for 36 h. Data shown are representative of three independent experiments. Scale bar = 1 μm.

*Hp *has long been known to transform into the coccoid form under unfavorable conditions, including exposure to atmospheric O_2 _levels. We examined the morphology of *Hp *grown under various levels of O_2 _and CO_2 _by field emission-scanning electron microscopy (FE-SEM) (Figure 3). The spiral form of *Hp *cells was observed at 12 h after inoculation, regardless of gas conditions. However, cultures grown under 8% O_2 _in the absence of CO_2 _also contained a significant number of coccoid *Hp *cells; at 36 h, most of the cells had transformed into U-shaped or coccoid cells. Under 20% O_2 _without CO_2_, most cells had very long spiral forms (mean length, 4.5 μm) at 12 h, but more than 60% of the cells were U-shaped, rounded, or coccoid at 36 h. These results indicate that high O_2 _levels delay *Hp *transformation into coccoid forms. Under CO_2_, most cells were spiral-shaped regardless of O_2 _tension at 12 h; however, at 36 h cells grown under 2% O_2 _began to convert to coccoid forms, whereas those cultured under 8% or 20% O_2 _remained in the unstressed spiral form. We noted numerous outer membrane vesicles (OMVs) at 12 h (mid-log phase) on the rugged surface of bacteria cultured under 8% or 20% O_2 _in the presence of 10% CO_2_, but OMVs were not observed and the cell surface became smooth at 36 h (stationary phase), suggesting that numerous changes occurred on cell surface structures during phase transition.

**Figure 4:**
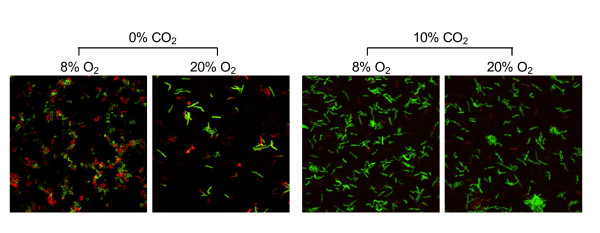
**Lack of CO_2 _induces coccoid transformation of *HP *cells**. *Hp *26695 was cultured in liquid medium for 12 h under various gas conditions. After staining with membrane-permeant SYTO 9 (green) and membrane-impermeant PI (red), cells were visualized by confocal microscopy. Data shown are representative of five independent experiments.

Next, we evaluated *Hp *cell membrane integrity under various gas conditions with membrane-permeant and membrane-impermeant fluorescent dyes (Figure 4). Live/dead cell staining with SYTO 9 and propidium iodide (PI) showed that, after 12 h of CO_2 _deprivation, many cells lost cytoplasmic membrane integrity under the microaerobic condition. At 36 h, these microaerobic cultures contained only U-shaped, coccoid, and aggregated forms that had lost membrane integrity (data not shown). In contrast, 20% to 30% of the cells in the culture grown under 20% O_2 _without CO_2 _retained spiral or bacillary forms with intact membranes at 12 h and may have been viable. This result is consistent with the viable counts of *Hp *in Figure 1A. In the presence of CO_2_, most cells remained spiral or rod-shaped with intact membranes regardless of O_2 _concentration. Along with FE-SEM findings, these results indicate that high CO_2 _tension is required for *Hp *survival and growth, and in the absence of CO_2_, aerobic conditions support *Hp *cell survival better than microaerobic conditions.

### *Hp *uses fermentation under microaerobic conditions but not under aerobic conditions

**Figure 5:**
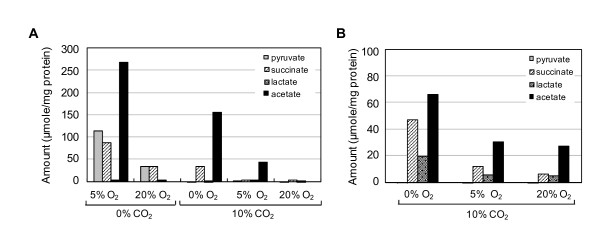
**Accumulation of fermentation products in culture media of *Hp *cells grown under low O_2 _levels**. *Hp *26695 was cultured in liquid medium for 36 h under various gas conditions with adding the appropriate gas mixture every 12 h (A) or without adding more gas (B). The culture medium was harvested and analyzed for organic acids by HPLC. The organic acid concentrations secreted from bacteria were calculated by subtracting each organic acid level in media control, and converted into μmol secreted per mg bacterial protein. Data shown in A and B are representative of three and two independent experiments, respectively.

Because our results indicated that *Hp *is not microaerophilic at high cell densities and grows better under aerobic conditions, we assessed *Hp *energy metabolism by measuring metabolites under microaerobic or aerobic conditions. In the initial culture media, the glucose level was 2.5 mM but became undetectable in the media of cultures grown under 8% or 20% O_2 _with 10% CO_2_, where bacterial growth was significantly higher, indicating glucose consumption (data not shown). Acetate was the major organic acid product in cultures grown under anaerobic and microaerobic conditions, followed by pyruvate and succinate (Figure 5A). Cultures grown under 20% O_2 _in the presence of CO_2 _secreted little organic acid into the medium, indicating that *Hp *does not use fermentation to generate ATP under this condition. Pyruvate is a pathway intermediate and not a typical fermentation product. It was detected only in the media of cultures grown without CO_2 _supply regardless of O_2 _level, which suggested that pyruvate was released from dead cells grown under CO_2_-depleted conditions. For this experiment, we refilled the flasks with the appropriate gas mixture every 12 h to supply CO_2_; therefore, exposure of cultures to air may have affected our results. To avoid exposure to atmospheric O_2_, we then cultured cells for 36 h without adding gas. The levels of acetate, succinate, and lactate were higher in all three cultures and were inversely associated with the initial O_2 _levels (Figure 5B). Oxygen depletion in the closed flasks may account for the higher fermentation rates observed in this experiment, even in the culture grown under 20% O_2 _tension. These data suggest that *Hp *uses fermentation under microaerobic conditions but aerobic respiration under aerobic conditions.

### Maintenance of intracellular pH is not the sole reason for the CO_2 _requirement

*Hp *is a neutralophile with a bioenergetic profile suited for growth at neutral pH [34]. However, *Hp *resides in a highly acidic environment and has therefore developed systems for acclimation. CO_2 _produced by urease is essential for the viability of *Hp *in the acidic environment; the periplasmic α-carbonic anhydrase (CA) converts the CO_2 _to bicarbonate, which buffers the periplasm [40]. We hypothesized that the CO_2 _requirement for *Hp *survival and growth may be due to reasons other than maintenance of internal pH. We tested this possibility by assessing changes in cytoplasmic and periplasmic pH during the culture of *Hp *cells grown in the absence or presence of CO_2_.

**Figure 6:**
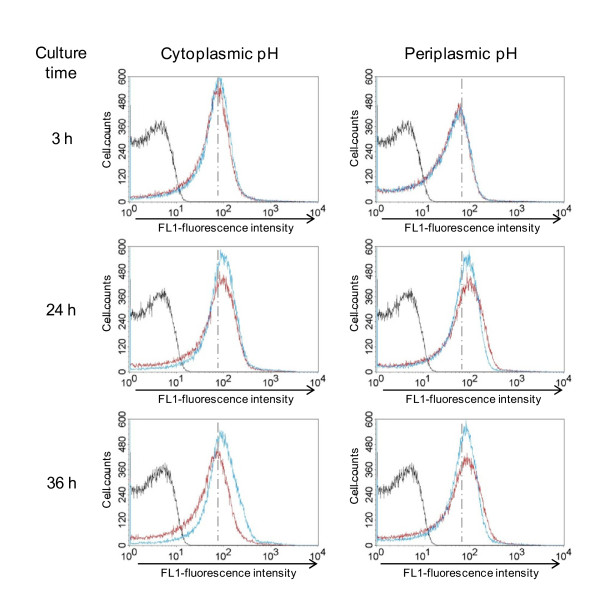
**CO_2 _deprivation does not cause changes in cytoplasmic or periplasmic pH until 24 h**. *Hp *26695 was inoculated into liquid medium containing the pH-sensitive fluorescent dye BCECF free acid or BCECF-AM, and cultured under 20% O_2 _tension in the absence (blue line) or presence (red line) of 10% CO_2_. An aliquot of each culture was taken at the indicated time points and analyzed by flow cytometry. Unstained *Hp *cells are shown for comparison (black line). Increase in fluorescence intensity represents higher pH. Data shown are representative of two independent experiments.

*Hp *26695 cells were cultured in liquid medium containing the pH-sensitive inner membrane-permeant fluorescent dye BCECF-AM to determine cytoplasmic pH and with the inner membrane-impermeant BCECF free acid to determine periplasmic pH. The cultures were grown under 20% O_2 _tension in the absence or presence of 10% CO_2 _and then analyzed by flow cytometry (Figure 6). Rapid alkalization of the culture medium was observed in the absence of CO_2_, which inhibited growth (data not shown); therefore, we buffered the liquid medium (pH 6.3). Subsequently, the culture medium pH remained below 7.3 until 36 h after inoculation, irrespective of gas conditions (Figure 1A). The absence of CO_2 _did not affect cytoplasmic or periplasmic pH until 24 h after inoculation, when the cytoplasmic pH of the cells cultured without CO_2 _began to rise, reflecting the cell death observed in the live/dead cell staining (Figure 4). On the basis of these findings, we concluded that CO_2 _deprivation does not cause changes in cytoplasmic or periplasmic pH and that the maintenance of pH homeostasis alone cannot account for the high CO_2 _requirement for *Hp *growth.

### Accumulation of intracellular ATP in *Hp *cells deprived of CO_2_

**Figure 7:**
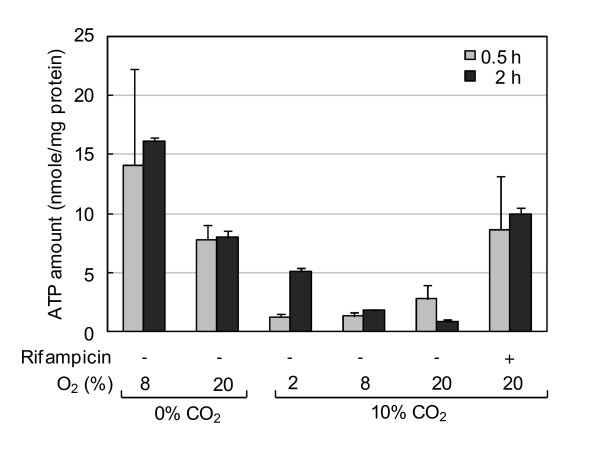
**Increased intracellular ATP levels in *Hp *deprived of CO_2 _or treated with rifampicin**. *Hp *26695 was cultured in liquid media for 0.5 or 2 h under various gas conditions in the absence or presence of rifampicin. Intracellular ATP levels were determined by luciferase assay. Results are expressed as mean ± SD of triplicate cultures. Data shown are representative of five experiments performed without rifampicin and two experiments performed with rifampicin.

To determine whether CO_2 _deprivation affects the intracellular energy state of *Hp*, we determined intracellular ATP levels of cells grown in the absence or presence of CO_2_. *Hp *26695 cells were cultured under 20% O_2 _with or without CO_2 _for 0.5 or 2 h, and intracellular ATP levels were determined by luciferase assay (Figure 7). The ATP level of cells deprived of CO_2 _was 4 to 8 times higher than that of cells grown under 10% CO_2_. In the absence of CO_2_, the ATP level of cells grown under the microaerobic condition was higher than that of cells grown under the aerobic condition. O_2 _tension also tended to be inversely correlated to the ATP level in the presence of CO_2_. Treatment of cells with rifampicin, which inhibits gene transcription, also increased ATP levels. Intracellular ATP levels appeared inversely associated with growth rate, and therefore its accumulation may be due to cessation of biosynthesis processes.

### Lack of CO_2 _induces the stringent response in *Hp *cells

**Figure 8:**
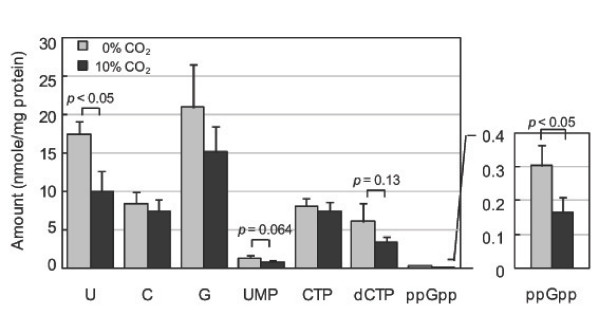
**Increased intracellular ppGpp levels in *Hp *cells in the absence of CO_2_**. *Hp *26695 was cultured in liquid media for 1 h under an aerobic condition in the absence or presence of 10% CO_2_, and intracellular nucleotide levels were determined by HPLC analysis. Results are presented as mean ± SD of values obtained from triplicate cultures. Data shown are representative of three independent experiments.

The stringent response, which is broadly conserved among bacterial species, enables bacteria to adapt to nutrient stress conditions [41,42]. It is characterized by the rapid accumulation of the alarmones guanosine tetraphosphate (ppGpp) and guanosine pentaphosphate (pppGpp) and the downregulation of nucleic acid and protein synthesis. *Hp *initiates the stringent response upon nutrient and pH downshift [41]. To determine whether CO_2 _deprivation induces the stringent response in *Hp*, we assessed intracellular nucleotide pools by high-performance liquid chromatography (HPLC) (Figure 8). In the presence of 10% CO_2_, intracellular ppGpp level was 0.17 nmol per mg bacterial protein, but pppGpp was not detected. Lack of CO_2 _significantly increased the ppGpp level, suggesting induction of the stringent response. We noted that uracil was also significantly higher in cells cultured without CO_2_. Furthermore, levels of uridine 5'-monophosphate (UMP) and deoxycytidine triphosphate (dCTP), but not cytosine or cytidine-5'-triphosphate (CTP), appeared higher in these cells, although the differences were not significant.

## Discussion

*Hp *has long been considered a microaerophile that requires O_2 _for growth but is highly sensitive to atmospheric O_2 _levels. In the present study, however, we demonstrate that atmospheric O_2 _tension does not kill *Hp *cells but promotes growth of cells when inoculated at high density, and *Hp *is unique in that it absolutely requires high CO_2 _tension for optimal growth and long-term survival. Eliminating the need to remove O_2 _makes it considerably easier to culture *Hp *in the laboratory.

Bury-Moné et al. reported that *Hp *strains showed similar growth profiles under aerobic and microaerobic conditions. However, when cells were inoculated in medium containing 0.2% β-cyclodextrin to low density (10^7 ^CFU/ml), growth was not detected under 15% O_2 _and 6% CO_2 _(generated with CO_2 _Gen gas packs) [31]. In contrast, we found that atmospheric O_2 _tension did not kill *Hp *cells but did prolong the lag period of cultures inoculated at low cell density (3 × 10^4 ^CFU/ml). The conflicting results may have been due to different experimental conditions. We used 10% CO_2 _to culture *Hp*, whereas the previous study used 6% CO_2_. Culture medium pH may increase faster under lower CO_2 _levels than under 10% CO_2_, thereby inhibiting bacterial growth, particularly under 20% O_2_. Further, because the lag period of low-density cultures is prolonged under 20% O_2_, the culture period in the previous study may have been insufficient to detect growth.

Bury-Moné et al. investigated whether growth inhibitory factors played a role in the lack of *Hp *growth under aerobic conditions. In one experiment, *Hp *cells were inoculated at high or low densities in two compartments separated by a membrane that stopped bacteria moving between them but allowed the exchange of metabolites, chemical compounds, and macromolecules, and cultured under aerobic or microaerobic conditions. In another experiment, a freshly inoculated culture was supplemented with culture medium in which a high-density or low-density culture had grown. Neither experiment revealed effects of inhibitory factors [31].

In the present study, we found that the effect of O_2 _on *Hp *growth was dependent on inoculum size: aerobic conditions inhibited growth in low-density cultures but induced growth in high-density cultures. Conversely, under low O_2 _tension, low-density cultures grew faster than high-density cultures. In the present study, HPLC analysis of *Hp *metabolites revealed higher levels of acetate, succinate, and lactate at lower O_2 _tensions. These results are consistent with previous reports that *Hp *utilizes aerobic respiration or fermentation, depending on environmental O_2 _levels, suggesting a possibility that *Hp *is a facultative anaerobe. On the basis of these data, we presumed that it is more efficient for a low-density culture to generate ATP by fermentation rather than by aerobic respiration. In *Escherichia coli*, enzymes involved in the tricarboxylic acid (TCA) cycle are significantly downregulated (2- to 10-fold) and fermentation enzymes are highly upregulated (>10-fold) when glucose is used as a carbon source under microaerobic conditions; the reverse is true under aerobic conditions [43]. Likewise, in *Hp*, fermentation enzyme activity would be expected to be lower under 20% O_2 _than under 2% or 8% O_2_. In addition, we observed that *Hp *produced more organic acids in the absence of CO_2 _than in the presence of CO_2 _(Figure 5C), suggesting that CO_2 _is important for efficient aerobic respiration in *Hp *cells, probably for enzyme induction.

CO_2 _is involved in a wide range of biological processes, and the addition of CO_2 _has been shown to shorten the lag period of bacterial cultures [44]. *Hp *requires high level of CO_2 _for its growth and generates a large amount of CO_2 _through urease activity. The shaking of cultures during incubation dissipates metabolic CO_2_, thus *Hp *growth would be greatly influenced by inoculating cell density, especially under aerobic conditions. We tested this possibility by supplementing a culture inoculated at low density (3 × 10^4 ^CFU/ml) with bicarbonate; however, bicarbonate did not increase the growth rate (data not shown).

Another possible explanation for the growth inhibiting effect of O_2 _is the bacterial signaling system known as quorum sensing, which monitors cell population density [45]. Bacteria release low molecular-weight autoinducers that accumulate in the environment; at threshold concentrations, these signaling molecules induce the coordinated expression of target genes in the population. *Hp *has been shown to possess a quorum-sensing system [46], and autoinducer 2 appears to regulate motility and flagella morphogenesis [47]. In *Pseudomonas aeruginosa*, expression of the quorum-sensing regulatory protein LasR is regulated by iron and O_2 _[48]. It is not known whether O_2 _concentration serves as an environmental signal for monitoring cell density in *Hp*. The precise mechanism for the growth inhibition by high O_2 _levels is under investigation.

Numerous studies have been carried out to elucidate *Hp *physiology under oxidative stress, including studies of morphology, gene expression, and protein expression. However, in some of these experiments, *Hp *was cultured under atmospheric O_2 _tension without supplemental CO_2 _[29,49-51]. Therefore, coccoid transformation and subsequent cellular changes may have resulted, at least in part, from CO_2 _deprivation rather than oxidative stress.

A unique feature of *Hp *is its transformation to coccoid form under stress conditions. Coccoid transformation was thought to be a passive conversion that eventually leads to cell death [49]. However, several recent reports have suggested that coccoid transformation is an active process that allows *Hp *to adapt to its environment [52-54]. In the present study, CO_2 _deprivation induced coccoid formation, but this morphological transformation was delayed in cells cultured under high O_2 _tension, supporting the view that coccoid transformation of *Hp *is not a passive process but an active energy-consuming process.

In this study, we observed that actively growing cells, but not those at a stationary phase, produce OMVs, which are discrete, closed outer membrane blebs produced by gram-negative bacteria, especially pathogenic strains [55]. They are believed to serve as secretory vesicles that transmit virulence factors to host cells. OMVs are released by actively growing cells, and their maximal production occurs at the end of log phase in *E. coli, Vibrio cholerae*, and *Brucella melitensis *[56-58]. *Hp *OMVs are involved in biofilm formation in vitro and deliver VacA cytotoxin to gastric epithelium [59,60]. They induce growth arrest and IL-8 production by gastric epithelial cells, which have been associated with gastritis caused by *Hp *infections [61,62], and also enhances the carcinogenic potential of *Hp *[63]. Taken together, these reports and results obtained in the present study indicate the higher virulence of actively growing *Hp *cells, which are able to damage host cells through toxin delivery.

In the present study, cultivation of *Hp *cells in the absence of CO_2 _increased intracellular ppGpp levels, suggesting induction of the stringent response, which induces a global alteration in cellular transcription and indirectly activates genes involved in amino acid biosynthesis [42,64]. Many factors induce the stringent response, but nutrient stress from amino acid starvation has been the best studied. Induction of the stringent response by CO_2 _deprivation has also been reported in *Campylobacter jejuni*, a capnophilic microaerophile that is closely related to *Hp *[65].

The bicarbonate concentration of gastric juice is approximately 25 mM [66]. *Hp *generates additional CO_2 _via the breakdown of urea, thereby increasing bicarbonate levels. In fact, the gastric CO_2 _levels in *Hp*-positive volunteers were significantly higher than those of *Hp*-negative subjects [67]. The affinity for CO_2 _may thus be related to its ecological niche, which may have lead to adaptation and eventually dependency on high CO_2 _concentrations. *Hp *shows chemotactic responses towards high CO_2 _concentration in vitro [68]. Elevated levels of CO_2_/bicarbonate serve as a signal of the host environment and often increase the expression of diverse virulence factors [69,70]; however, the association between CO_2 _and virulence in *Hp *remains to be determined.

## Conclusions

In this manuscript, we showed that *H. pylori *may be a capnophilic aerobe whose growth is promoted by atmospheric oxygen levels in the presence of 10% CO_2_. Our data also suggest that buffering of intracellular pH alone cannot account for the CO_2 _requirement of *H. pylori *and that CO_2 _deprivation initiates the stringent response in *H. pylori*. Our findings may provide new insight into the true physiology of this fastidious human pathogen and contribute to understanding of its pathogenic mechanism(s).

## Abbreviations

BB: brucella broth; BCECF: 2,7-bis-(2-carboxyethyl)-5-carboxyfluorescein; BCECF-AM: 2,7-bis-(2-carboxyethyl)-5-carboxyfluorescein acetoxymethyl ester; CA: carbonic anhydrase; CFU: colony forming unit; DIG: digoxigenin; FE-SEM: field emission-scanning electron microscopy; *Hp*: *Helicobacter pylori*; HPLC: high performance liquid chromatography; NBCS: new born calf serum; OMVs: outer membrane vesicles; PBS: phosphate-buffered saline; PI: propidium iodide; ppGpp: guanosine tetraphosphate; pppGpp: guanosine pentaphosphate; rRNA: ribosomal RNA; SD: standard deviation.

## Competing interests

The authors declare that they have no competing interests.

## Authors' contributions

SAP participated in the design of the study, carried out the experiments, analyzed the data and drafted the manuscript. AK participated in the EM studies, part of the bacterial growth analysis. NGL conceived of the study and participated in its design, data analysis, coordination and writing of the manuscript. All authors read and approved the final manuscript.
